# Transarterial chemoembolization combined with apatinib with or without PD-1 inhibitors in BCLC stage C hepatocellular carcinoma: A multicenter retrospective study

**DOI:** 10.3389/fonc.2022.961394

**Published:** 2022-09-30

**Authors:** Wei-Li Xia, Xiao-Hui Zhao, Yuan- Guo, Guang-Shao Cao, Gang Wu, Wei-Jun Fan, Quan-Jun Yao, Shi-Jun Xu, Chen-Yang Guo, Hong-Tao Hu, Hai-Liang Li

**Affiliations:** ^1^ Department of Minimal-Invasive Intervention, The Affiliated Cancer Hospital of Zhengzhou University&Henan Cancer Hospital, Zhengzhou, China; ^2^ Department of Intervention, Henan Provincial People’s Hospital, Zhengzhou, China; ^3^ Department of Interventional Radiology, The First Affiliated Hospital of Zhengzhou University, Zhengzhou, China; ^4^ Department of Minimally Invasive Interventional Radiology, Sun Yat-sen University Cancer Center, Guangzhou, China

**Keywords:** hepatocellular carcinoma, transarterial chemoembolization, apatinib, PD-1 inhibitors, propensity score matching, random survival forest (RSF)

## Abstract

**Objective:**

We evaluated the efficacy and safety of transarterial chemoembolization (TACE) combined with apatinib plus PD-1 inhibitors (TACE-AP) compared with TACE combined with apatinib (TACE-A) in patients with advanced hepatocellular carcinoma (HCC) and to explore the prognostic factors affecting patient survival.

**Methods:**

Data from patients with unresectable HCC who received TACE-AP or TACE-A from December 2018 to June 2021 were collected retrospectively. The main outcome of the study was overall survival (OS) and prognostic factors affecting survival, while the secondary outcomes were progression-free survival (PFS), the objective response rate (ORR), and treatment-related adverse events (TRAEs). Propensity score matching (PSM) analysis was used to reduce patient selection bias, and the random survival forest (RF) model was employed to explore prognostic factors affecting patient survival.

**Results:**

We enrolled 216 patients, including 148 and 68 patients in the TACE-A and TACE-AP groups, respectively. A total of 59 pairs of patients were matched using PSM analysis. Before and after PSM, the OS, PFS, and ORR in the TACE-AP group were significantly higher than in the TACE-A group (before, OS: 22.5 months vs. 12.8 months, P < 0.001; PFS: 6.7 months vs. 4.3 months, P < 0.001; ORR: 63.2% vs. 34.5%, P < 0.001; after, OS: 22.5 months vs. 12.0 months, P < 0.001; PFS: 6.7 months vs. 4.3 months, P < 0.001; ORR: 62.7% vs. 30.5%, P = 0.003). Multivariate Cox regression and RF models before and after PSM analysis revealed that the main prognostic factors affecting survival were tumor number, portal vein tumor thrombus (PVTT) invasion, alpha-fetoprotein (AFP) levels, total bilirubin (TBIL) level, and treatment. There was no significant difference in TRAEs between the two groups (P > 0.05).

**Conclusion:**

Compared with TACE-A, TACE-AP significantly improved OS, PFS, and ORR in patients with advanced HCC. The number of tumors, PVTT invasion, AFP levels, TBIL level, and treatment were significant prognostic factors associated with patient survival. All observed TRAEs were mild and controllable.

## Introduction

Liver cancer is the sixth most common malignancy and the fourth leading cause of tumor-related death worldwide, with close to half of global patients with hepatocellular carcinoma (HCC) being from China (1, 2). HCC is the most common pathologic type of primary liver cancer, with the characteristics of insidious onset and high malignancy. Most patients are diagnosed with HCC in the middle and late stages, contributing to a poor disease prognosis (3, 4).

Transarterial chemoembolization (TACE) is the most effective treatment option for patients with inoperable liver cancer (5). However, previous studies found that the efficacy of TACE attenuates over time and aggravates liver damage. Tumors are in a hypoxic environment after TACE, which can upregulate the expression of angiogenic factors and increase the risk of recurrence and metastasis of HCC (6). These factors result in unsatisfactory long-term efficacy of TACE in patients with HCC. Previous studies showed that the survival rate of patients is greater when combined therapy is used as early as possible after TACE (7).

Apatinib is a small-molecule multi-target tyrosine kinase inhibitor that can inhibit tumor angiogenesis by selectively inhibiting the tyrosine kinase activity of vascular endothelial growth factor receptor 2 (VEGFR-2). Previous studies showed that apatinib combined with TACE can significantly reduce tumor volume, inhibit tumor revascularization, and prolong patient survival (8). Immune checkpoint inhibitors (PD-1 inhibitors), which can reverse immune failure, are effective in the treatment of advanced HCC when used concomitantly with TACE (9). Compared with monotherapy, the combination of TACE, PD-1 inhibitors, and an antiangiogenic agent showed excellent antitumor activity and significantly improved patient survival (10).

Currently, TACE combined with apatinib and PD-1 inhibitors shows great potential for the treatment of advanced HCC (11). However, no control studies of TACE combined with apatinib (TACE-A) and TACE combined with apatinib and PD-1 inhibitors (TACE-AP) have been reported to date. This retrospective multicenter study compared the efficacy and safety of TACE-A and TACE-AP to treat patients with advanced HCC, and we analyzed the prognostic factors affecting patient survival using a random survival forest (RF) model.

## Materials and methods

### Patients

Clinical records for patients with HCC treated with TACE-A or TACE-AP from December 2018 to June 2021 were collected and analyzed retrospectively. The study was approved by the Ethics Committee of the Affiliated Cancer Hospital of Zhengzhou University and the Henan Cancer Hospital review board (approval number 2019198). The study was retrospective in nature; therefore, all requirements for informed consent were waived, and anonymous analyses of extracted data were performed.

**Figure 1:**
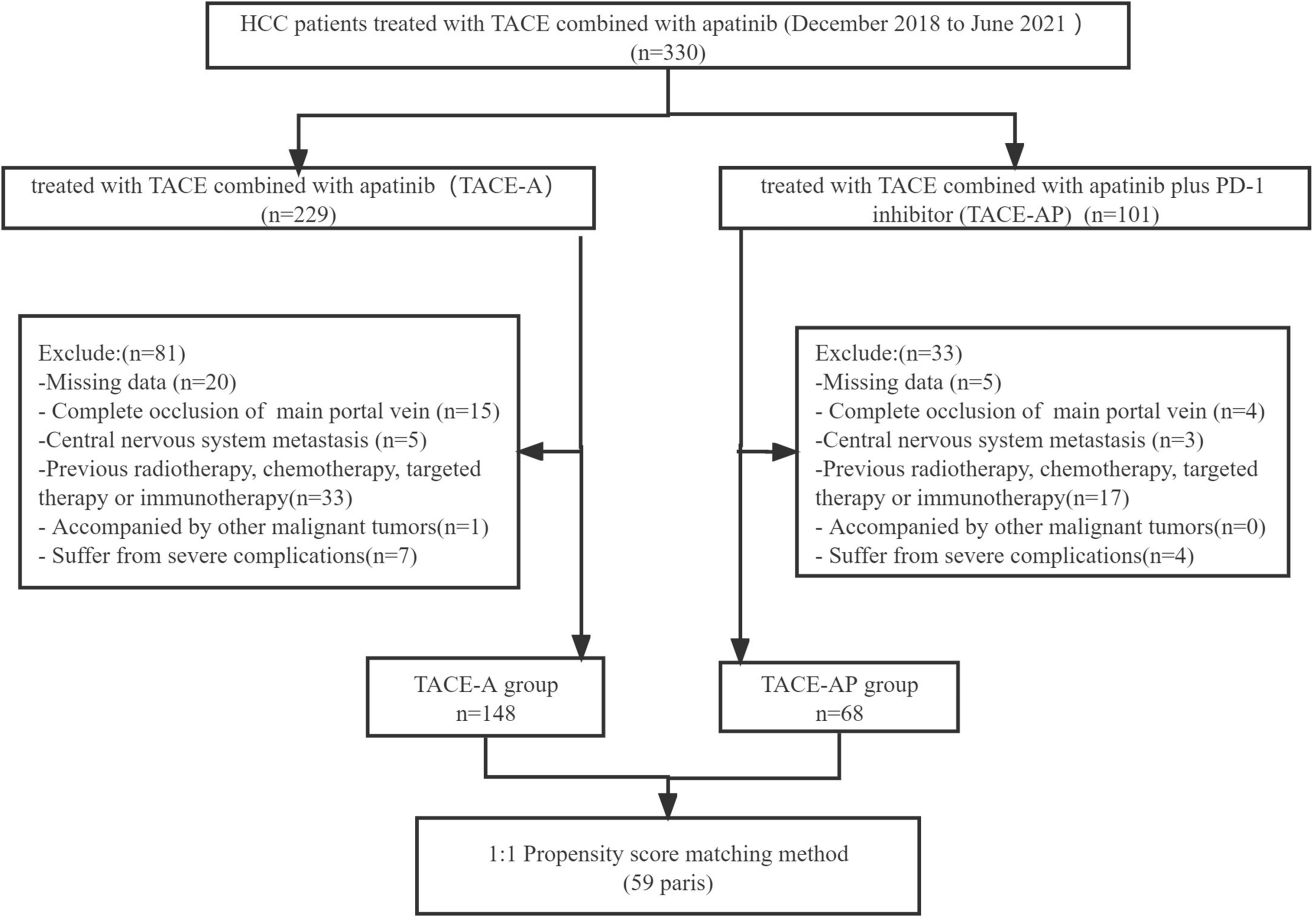
Flow diagram of patient screening.

Patients who met the following criteria were included in the study: (1) aged between 18 and 75 years; (2) diagnosed with HCC based on pathological data or using the non-invasive criteria outlined by the American Association for the Study of Liver Diseases; (3) categorized as Barcelona Clinic Liver Cancer (BCLC) tumor stage C; (4) Child-Pugh grade A or B; (5) Eastern Cooperative Oncology Group (ECOG) ≤ 1; and (6) platelet counts ≥ 60 × 10^9^/L. The exclusion criteria were: (1) complete occlusion of the main portal vein or invasion of the superior mesenteric vein; (2) central nervous system metastasis; (3) previous radiotherapy, chemotherapy, targeted therapy, or immunotherapy; (4) other malignant tumors; and (5) the presence of severe complications, including severe cardiac, pulmonary, renal, or coagulation disorders (**Figure 1**).

### TACE procedure

As reported previously, the TACE procedure remained consistent across the centers (12). All TACE procedures were performed by at least two experienced interventional radiologists using the traditional femoral artery approach under local anesthesia. 5F RH catheters (Terumo, Tokyo, Japan) were first used for routine angiography, and then microcatheters (Terumo) were used for super-selective arterial catheterization to enter the blood supply branch of the tumor. A mixed solution containing lipiodol (Laboratoire Guerbet, Paris, France) and doxorubicin (Haizheng Pharmaceutical, Taizhou, China) was injected into the tumor-feeding vessels, followed by injection with gelatin sponge particles of 500–700 µm (ALICON Pharmaceutical, Hangzhou, China) to supplement embolization until the blood flow nearly ceased. The dose of doxorubicin was 50–70 mg, while that of lipiodol was 5–20 mL. The individualized doses were adjusted according to the patient’s tumor number and size, liver function, blood vessel distribution, and body surface area.

### Administration of apatinib and PD-1 inhibitors

After three days of TACE, patients in the TACE-A group received oral apatinib (Hengrui Pharmaceutical, Lianyungang, China) at a dose of 250 mg/day, and those in the TACE-AP group received the same dose of apatinib and an intravenous injection of PD-1 inhibitors (200 mg) every three weeks until intolerable toxicity or disease progression. The administered PD-1 inhibitors included sintilimab (Innovent Pharmaceutical, Suzhou, China), tislelizumab (BeiGene Pharmaceutical, Shanghai, China), pembrolizumab (Merck, Kenilworth, NJ, USA), and camrelizumab (Hengrui Pharmaceutical, Lianyungang, China).

When patient adverse events were equal to or greater than grade 3 and determined to be related to apatinib, apatinib was administered every other day, and its administration was suspended if the adverse events continued after the dose adjustment. Nevertheless, the dose was restored to 250 mg/day if the adverse events were relieved or eliminated. If the adverse events were determined to be related to PD-1 inhibitors, the medication was suspended and was not resumed until the adverse events had resolved. Generally, patient follow-up should be performed every 4–6 weeks after TACE. From the extracted data, the follow-up included chest computed tomography (CT) and liver multiphase enhanced magnetic resonance imaging (MRI), routine blood tests, and liver and renal function tests. When MRI results showed recurrence or residual activity of the intrahepatic tumor, TACE should be repeated if the patient has good liver function test results. Herein, patients received apatinib continuously before TACE was repeated, and the treatment was interrupted for three days after TACE was repeated.

The original treatment regimen was discontinued if intolerable toxicity or disease progression occurred during the follow-up period. Following discussions between the multidisciplinary team and according to the patient’s wishes, all patients were treated under the same principles as follows: PD-1 inhibitors (for patients in the TACE-A group), a second-line targeted drug (regorafenib), radiotherapy (including radioactive iodine 125 seed implantation), or best supportive care. The main outcomes of this study were median overall survival (OS) and prognostic factors affecting survival, while the secondary outcomes were median progression-free survival (PFS), objective remission rate (ORR), and treatment-related adverse events (TRAEs).

### Evaluation criteria

The last follow-up of the study was conducted in April 2022. OS was defined as the time to death or last follow-up. According to the modified evaluation criteria for solid tumors (mRECIST), the patient’s imaging data were evaluated by two radiologists with intermediate titles or above, and the results were assessed as complete remission (CR), partial remission (PR), stable disease (SD), and disease progression (progressive disease, PD). Portal vein tumor thrombus (PVTT) invasion was stratified according to Cheng’s PVTT classification system: Type I, tumor thrombi involving segmental branches of the portal vein; Type II, tumor thrombi involving the right/left portal vein; and Type III, tumor thrombi involving the main portal vein and trunk. The adverse reaction grades were evaluated using the National Cancer Institute Standard for Adverse Events, version 3.0.

### PSM analysis

To balance the variables between the TACE-A and TACE-AP groups and to reduce patient selection bias, we performed PSM analysis using a 1:1 ratio to construct a balanced cohort with a caliper value of 0.1. Baseline variables included age, sex, BCLC stage, Child-Pugh grade, metastasis, PVTT, alpha-fetoprotein (AFP) levels, hepatitis B virus infection, and ECOG score, as well as total bilirubin, albumin, alanine aminotransferase, creatinine levels, and tumor size and number.

### Stochastic survival forest model

To avoid sample size loss caused by the PSM analysis, important factors affecting OS were further analyzed by constructing a stochastic survival forest model using variable importance (VIMP) and minimum depth methods. A VIMP value < 0 indicated that the variable reduced the accuracy of the prediction, while a VIMP value > 0 improved the prediction accuracy. The minimum depth method revealed the importance of each variable to the outcome event by calculating the minimum depth when running the final node.

### Statistical analysis

Categorical variables were expressed as percentages, calculated by the chi-squared test, while continuous variables were expressed as means ± standard deviation, calculated by the Student’s t-test. The difference in OS between the two groups was evaluated using the Kaplan-Meier method. The statistical significance of clinical characteristics was assessed by univariate analysis, and statistically significant variables were included in the analysis using multivariate Cox regression models to identify predictors associated with OS. The difference was considered statistically significant when P < 0.05. All analyses were performed using R statistical software (version 4.1.2; R Statistical Computing Foundation, Vienna, Austria; http://www.r-project.org/).

## Results

### Baseline characteristics

We screened 330 patients, 114 of which were excluded; thus, 216 (148 in the TACE-A group and 68 in the TACE-AP group) were included in this study. The administered PD-1 inhibitors were sintilimab (25 patients; 36.7%), tislelizumab (20 patients; 29.4%), camrelizumab (15 patients; 22.1%), and pembrolizumab (8 patients; 11.8%). **Table 1** lists the baseline characteristics of the patients before and after PSM analysis.

**Table 1:** Baseline characteristics of the two groups before and after PSM.

Variable	Before PSM					After PSM			
	Grading	TACE-A(n=148)	TACE-AP(n=68)	P value	*SMD*	TACE-A(n=59)	TACE-AP(n=59)	P value	*SMD*
Gender	Male	126 (85.1)	62 (91.2)	0.313	0.188	54 (91.5)	53 (89.8)	1.000	0.058
	Female	22 (14.9)	6 (8.8)			5 (8.5)	6 (10.2)		
Age	≤60	98 (66.2)	49 (72.1)	0.485	0.127	43 (72.9)	41 (69.5)	0.839	0.075
	>60	50 (33.8)	19 (27.9)			16 (27.1)	18 (30.5)		
Child-Pugh classification	A	130 (87.8)	65 (95.6)	0.124	0.284	57 (96.6)	56 (94.9)	1.000	0.084
	B	18 (12.2)	3 (4.4)			2 (3.4)	3 (5.1)		
PVTT	None	79 (53.4)	28 (41.2)	0.208	0.260	27 (45.8)	27 (45.8)	0.857	0.103
	Type I+II	50 (33.8)	31 (45.6)			22 (37.3)	24 (40.7)		
	Type III	19 (12.8)	9 (13.2)			10 (16.9)	8 (13.6)		
Metastasis	None	90 (60.8)	35 (51.5)	0.253	0.189	34 (57.6)	32 (54.2)	0.853	0.068
	Have	58 (39.2)	33 (48.5)			25 (42.4)	27 (45.8)		
AFP(ng/mL)	<400	87 (58.8)	42 (61.8)	0.791	0.061	32 (54.2)	35 (59.3)	0.710	0.103
	≥400	61 (41.2)	26 (38.2)			27 (45.8)	24 (40.7)		
HBV	None	47 (31.8)	29 (42.6)	0.161	0.277	22 (37.3)	22 (37.3)	1.000	<0.001
	Have	101 (68.2)	39 (57.4)			37 (62.7)	37 (62.7)		
ECOG Score	0	21 (14.2)	13 (19.1)	0. 470	0.133	12 (20.3)	12 (20.3)	1.000	<0.001
	1	127 (85.8)	55 (80.9)			47 (79.7)	47 (79.7)		
TBIL (g/L,mean±SD)		21.92±10.31	21.11±8.44	0.574	0.186	20.65±8.42	20.76±8.50	0.947	0.012
AlB (μmol/L,mean±SD)		37.25±4.91	38.01±6.90	0.358	0.126	38.11±4.56	37.72±7.12	0.729	0.064
ALT (U/L,mean±SD)		46.55±66.25	37.94±20.51	0.296	0.176	37.24±20.55	37.80±21.61	0.886	0.027
Cr (umol/L,mean±SD)		58.77±11.90	61.69±15.08	0.126	0.215	58.54±11.25	59.31±11.15	0.710	0.069
Number of liver tumors	1	25 (16.9)	17 (25.0)	0.373	0.201	11 (18.6)	11 (18.6)	1.000	<0.001
	2	76 (51.4)	31 (45.6)			28 (47.5)	28 (47.5)		
	≥3	47 (31.8)	20 (29.4)			20 (33.9)	20 (33.9)		
Maximum tumor diameter (mm,mean±SD)		77.08±45.79	70.09±37.57	0.272	0.167	69.41±44.64	68.48±37.66	0.903	0.023
Number of TACE		1.95 (1.16)	2.12 (1.52)	0.362	0.127	1.90 (1.12)	2.14 (1.54)	0.340	0.176

ECOG, Eastern Cooperative Oncology Group; AFP, alpha-fetoprotein; TBIL, total bilirubin; ALB, albumin; ALT, alanine aminotransferase; Cr, creatinine.

### Efficacy

**Figure 2:**
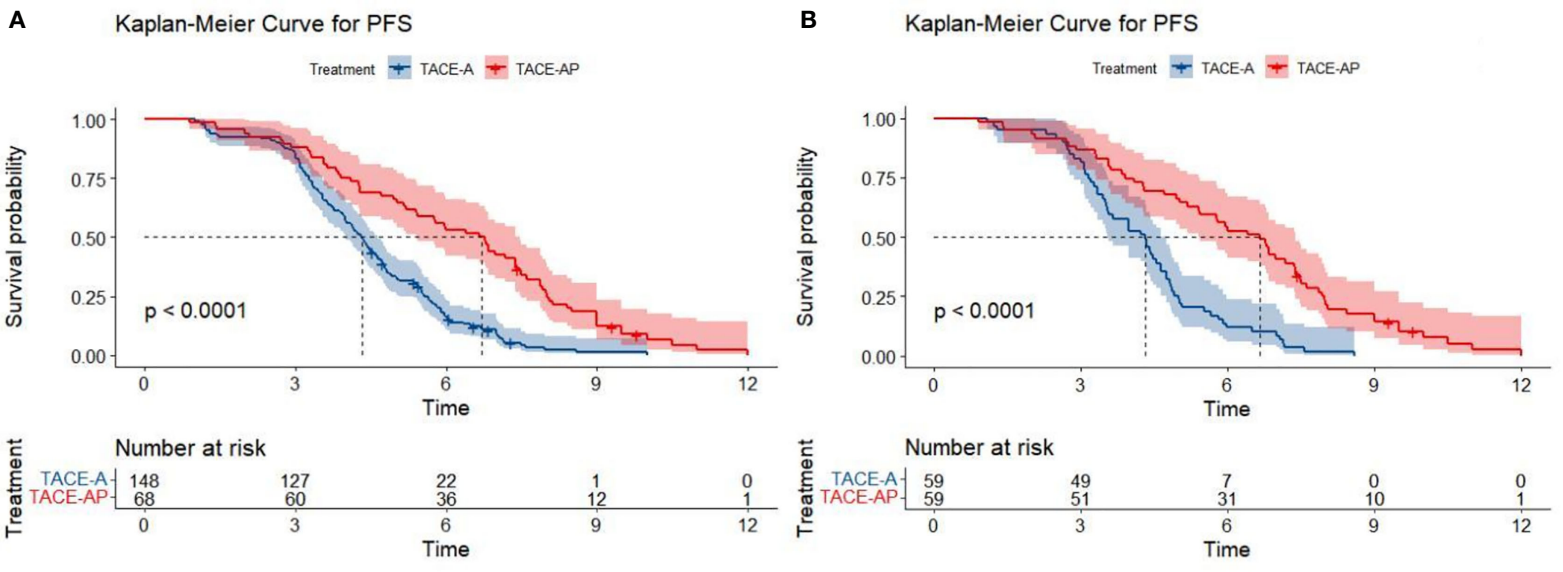
Kaplan-Meier curves for PFS in patients with advanced HCC treated with TACE-A or TACE-AP before **(A)** and after **(B)** PSM.

**Figure 3:**
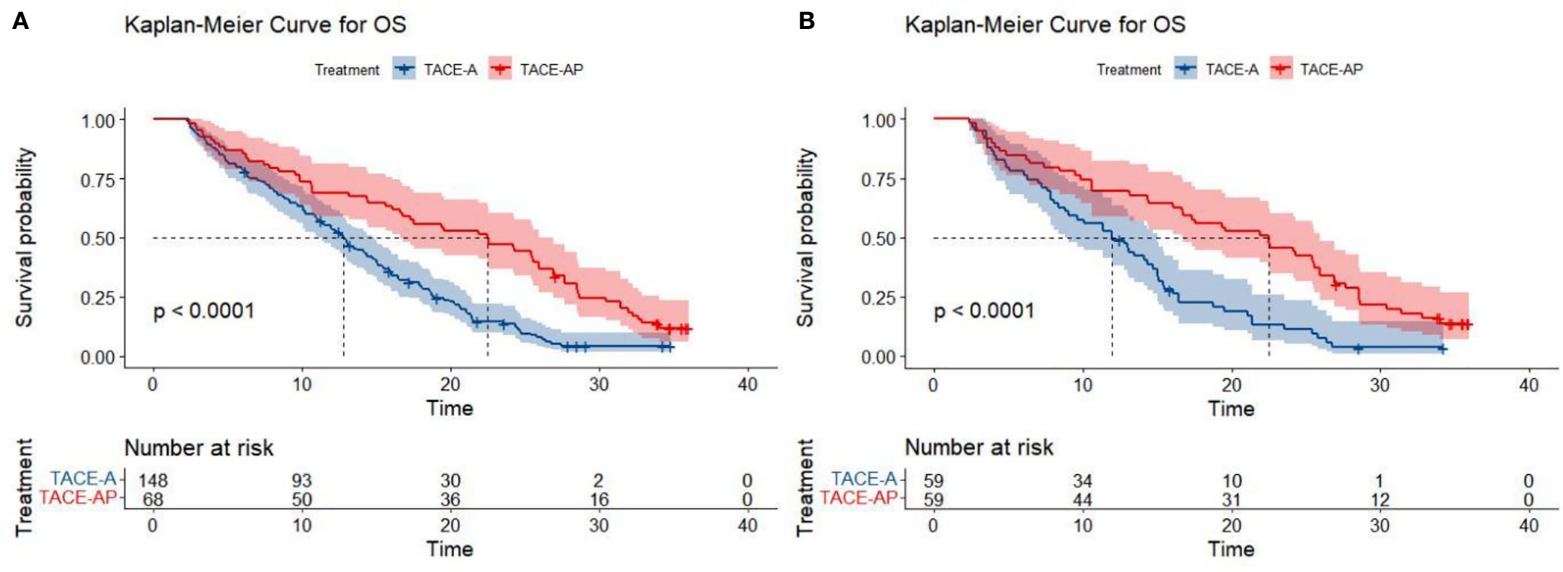
Kaplan-Meier curves for OS in patients with advanced HCC treated with TACE-A or TACE-AP before **(A)** and after **(B)** PSM.

At the end of the follow-up period (April 2022), the proportions of patients that reached the study endpoint in the TACE-A and TACE-AP groups were 94.6% (140/148) and 95.6% (65/68), respectively. Before PSM, the median PFS was 4.3 months (95% confidence interval [CI]: 3.9–4.6) and 6.7 months (95% CI: 5.4–7.5), and the median OS was 12.8 months (95% CI: 11.1–14.7) and 22.5 months (95% CI: 16.6–26.9) in the TACE-A and TACE-AP groups, respectively. There was a significant difference in the median PFS and median OS between the two groups (P < 0.001). After PSM, the median PFS was 4.3 months (95% CI: 3.6–4.7) and 6.7 months (95% CI: 5.4–7.4), and the median OS of the two groups was 12.0 months (95% CI: 8.8–15.0) and 22.5 months (95% CI: 16.6–26.0), respectively, and there was a significant difference between the groups (P < 0.001; **Figures 2**, **3**).

Before PSM, the ORR findings of the TACE-A and TACE-AP groups were 34.5% and 63.2%, respectively, with a significant difference between the two groups (P < 0.001). After PSM analysis, the ORR was 62.7% in the TACE-AP group and 30.5% in the TACE-A group, the former being significantly higher than the latter (P = 0.003). The tumor responses of all the patients are listed in **Table 2**.

**Table 2:** Tumor response before and after PSM analysis.

Tumor response	Before PSM		*P* value	After PSM		*P* value
	TACE-A (N = 148)	TACE-AP (N = 68)		TACE-A (N = 59)	TACE AP (N = 59)	
CR	14	9		3	8	
PR	37	34		15	29	
SD	65	13		29	12	
PD	32	12		12	10	
ORR (CR+PR)	34.5%	63.2%	<0.001	30.5%	62.7%	0.003

### Prognostic factors for OS

Univariate and multivariate Cox proportional hazard regression models were used to identify prognostic factors associated with OS (**Table 3**). Before PSM, univariate analysis showed that PVTT invasion, tumor number, treatment (TACE-A or TACE-AP), AFP, and total bilirubin (TBIL) level were the prognostic factors affecting OS (P < 0.05). The multivariate Cox analysis showed that distant metastasis (P = 0.048), PVTT (type = III; P = 0.002), AFP ≥ 400 ng/ml (P = 0.025), and multiple tumors (P < 0.001) were independent risk factors for OS, and TACE-AP treatment modality (TACE-A or TACE-AP; P < 0.001) were independent protective factors for OS. After PSM, the univariate analysis showed that PVTT invasion, tumor number, treatment modality, AFP, and TBIL were prognostic factors for OS (P < 0.05). The multivariate analysis showed that metastasis, PVTT invasion, tumor number, TBIL, and treatment modality were independent prognostic factors affecting OS (P < 0.05).

**Table 3:** Univariate and multivariate Cox proportional hazards regression model analysis of OS before and after PSM.

Variable	Before PSM				After PSM			
	Univariate analysis		Multivariate analysis		Univariate analysis		Multivariate analysis	
	HR (95% CI)	*P* value	HR (95% CI)	*P* value	HR (95% CI)	*P* value	HR (95% CI)	*P* value
Age	0.93 (0.68-1.26)	0.634			0.68 (0.44-1.05)	0.081		
Sex	0.78 (0.51-1.2)	0.257			0.66 (0.33-1.31)	0.235		
Child-Pugh classification	0.88 (0.52-1.49)	0.633			0.73 (0.27-1.99)	0.538		
Metastasis	1.15 (0.86-1.53)	0.347			1.49 (1.01-2.19)	0.043	1.62 (1.05 - 2.49)	0.028
PVTT Type I+II	1.22 (0.9-1.66)	0.209	1.13 (0.82 - 1.55)	0.448	1.3 (0.85-1.99)	0.233	0.79 (0.5 - 1.27)	0.337
PVTT Type III	3.63 (2.32-5.68)	<0.001	2.97 (1.87 - 4.7)	0.002	3.23 (1.79-5.82)	<0.001	2.1 (1.11 - 3.97)	0.023
AFP	1.87 (1.39-2.5)	<0.001	1.60 (1.18 - 2.16)	0.025	1.83 (1.24-2.7)	0.002	1.32 (0.85 - 2.04)	0.211
HBV	1.18 (0.87-1.59)	0.282			1.25 (0.84-1.87)	0.275		
ECOG score	0.76 (0.52-1.12)	0.162			0.64 (0.4-1.02)	0.06		
TBIL	1.02 (1.01-1.03)	0.005	1.01 (1 - 1.03)	0.106	1.03 (1.01-1.06)	0.002	1.03 (1.01 - 1.06)	0.005
ALB	0.99 (0.97-1.01)	0.468			1 (0.97-1.02)	0.766		
ALT	1 (1-1)	0.609			1 (1-1.01)	0.383		
Cr	0.99 (0.98-1)	0.090			0.99 (0.97-1)	0.129		
Maximum tumor diameter	1 (1-1.01)	0.188			1 (1-1.01)	0.610		
Number of liver tumors=2	2.75 (1.81-4.18)	<0.001	2.42 (1.56 - 3.76)	<0.001	2.64 (1.47-4.74)	0.001	2.58 (1.36 - 4.92)	0.004
Number of liver tumors≥3	3.21 (2.06-5.01)	<0.001	2.81 (1.77 - 4.45)	<0.001	3.58 (1.94-6.59)	<0.001	3.58 (1.83 - 7.00)	<0.001
TACE	0.93 (0.84-1.03)	0.182			0.88 (0.76-1.02)	0.082		
Treatment	0.45 (0.33-0.63)	<0.001	0.43 (0.31 - 0.61)	<0.001	0.43 (0.29-0.65)	<0.001	0.33 (0.21 - 0.53)	<0.001

Treatment: TACE-A and TACE-AP. All variables were included in a multivariate stepwise Cox regression analysis; only the variables with P < 0.05 in the final model are presented; HR, hazard ratio; CI, confidence interval.

### Construction of the RF model

The RF model was established based on baseline characteristics and treatment methods for 15 related factors using Harrell’s concordance index to calculate model accuracy. The consistency index is a common indicator used to evaluate survival analysis models. The value of the consistency index is positively correlated with the quality of the model. **Figure 3** shows a C-index of the model of approximately 0.705.

**Figure 4:**
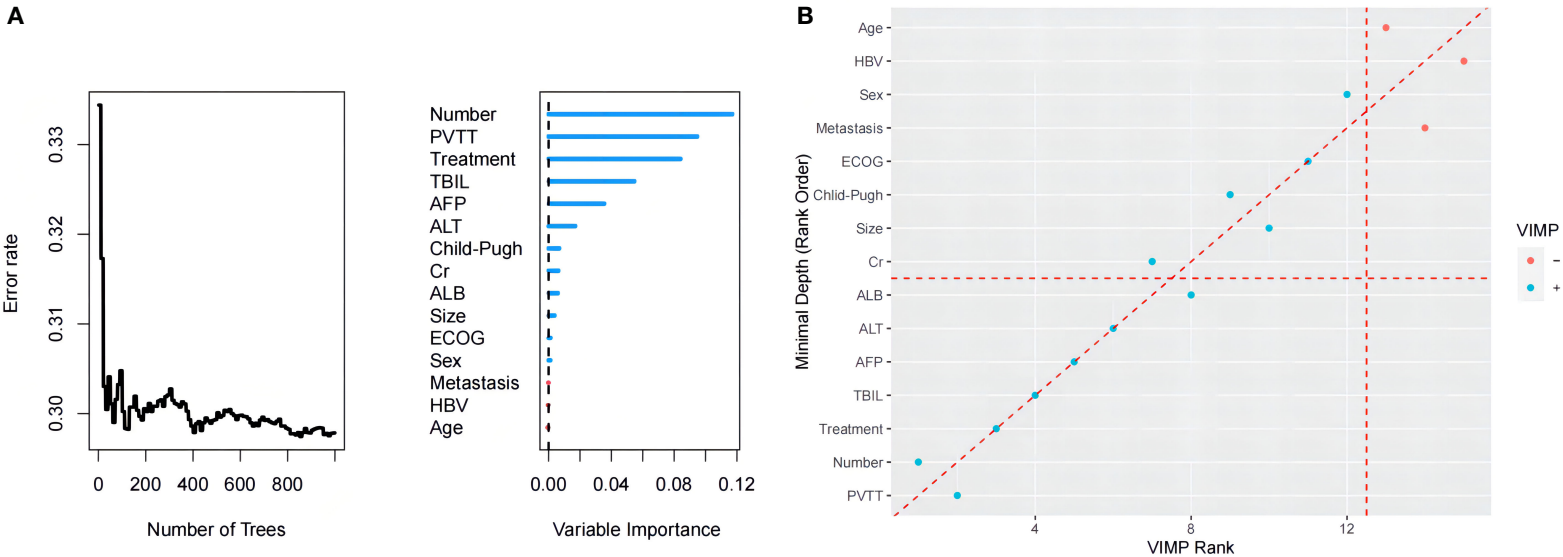
**(A)** Order of importance of the variables in the RF model for OS. **(B)** The blue point represents a VIMP value > 0, the red a value < 0; the point on the red diagonal dashed line represents the same ranking of the two methods on the variable, and the point higher than the diagonal dashed line represents its VIMP ranking. Higher points below the diagonal dotted line represent a higher minimum depth ranking.

The model generated 1000 binary survival trees by default. As depicted in **Figure 4A**, when the number of surviving trees increased to a certain value, the error rate curve leveled off (29.5%). **Figure 4B** shows the scatter plot of the two methods of the integrated VIMP and minimum depth method. The results show that AFP and TBIL were important variables that simultaneously met both conditions in PVTT invasion, tumor number, treatment (TACE-A or TACE-AP), and albumin.

### TRAEs

During the treatment and follow-up periods, the most common TRAEs in the two groups were post-embolization syndrome, including nausea and vomiting, pain, fatigue, and fever, which improved after symptomatic treatment. There was no significant difference in the incidence of TRAEs above grade 3 between the two groups (P > 0.05). All TRAEs were resolved after symptomatic treatment, drug dose reduction, or discontinuation. In the TACE-AP group, three patients (4.41%) developed hyperthyroidism, nine (13.23%) developed hypothyroidism, and one (1.47%) developed reactive cutaneous capillary endothelial proliferation, all of which were graded 1 or 2 and were relieved after symptomatic treatment. Immune-related pneumonia occurred in two cases (2.94%), of which one case reached grade 3, which gradually eased after stopping the PD-1 inhibitor. There were no deaths related to adverse reactions (**Table 4**).

**Table 4:** Treatment-related adverse events between TACE-A and TACE-AP groups.

AEs	TACE-A (N = 148)		TACE- AP (N = 68)	
	Any grade	≥ grade 3	Any grade	≥ grade 3
Fever	71(47.97)	2 (1.11)	31 (45.59)	0
Pain	61 (41.22)	5 (3.38)	28 (41.18)	2(2.94)
Nausea and Vomiting	45 (30.41)	0	21 (30.89)	0
Hand-foot skin reactions	65 (43.92)	4 (2.22)	28 (41.18)	3 (4.41)
Hypertension	48 (32.43)	2 (1.11)	25 (36.76)	1 (1.47)
Proteinuria	5 (3.38)	0	2 (2.94)	0
Fatigue	10 (6.76)	0	5 (7.35)	0
Hoarseness	5 (2.70)	0	1 (1.47)	0
Oral ulcer	8 (5.41)	0	2 (2.94)	0
Rash	7 (4.73)	0	1 (1.47)	0
Gastrointestinal hemorrhage	9 (6.08)	0	3 (4.41)	0
Gastrointestinal reaction	27 (17.56)	0	15 (22.06)	0
RCCEP	0	0	1 (1.47)	0
Hypothyroidism	0	0	9 (13.23)	0
Hyperthyroidism	0	0	3 (4.41)	0
Pneumonia	0	0	2 (2.94)	1 (1.47)
Myocarditis	0	0	1 (1.47)	0

RCCEP, Reactive cutaneous capillary endothelial proliferation.

## Discussion

TACE is one of the most effective treatments for advanced unresectable HCC (5, 13). However, due to disease heterogeneity and the limitations of TACE itself, the long-term effect of TACE is not guaranteed. The TACTICS study revealed that TACE combined with sorafenib significantly improved TACE efficacy. The results of several clinical trials on atlizumab showed that when used in patients with HCC, atlizumab- plus bevacizumab-treated patients had a longer OS and PFS than sorafenib or atlizumab alone. KEYNOTE 524 studies have suggested that pembrolizumab combined with lenvatinib can significantly improve the survival of patients with advanced HCC. These clinical trials confirmed that tyrosine kinase inhibitors combined with PD-1 inhibitors can confer survival benefits to patients with unresectable HCC. These results show that the combination of TACE, targeted therapy, and immunotherapy can improve therapeutic efficacy in patients with unresectable HCC (6, 14–16). A recent RESCUE trial demonstrated that apatinib combined with carilizumab showed better efficacy and safety as a first- or second-line treatment for advanced HCC, and it is worth noting that most patients in that study received local treatment, including TACE, after progression (17). Owing to these reports, we conducted a multicenter retrospective study of TACE combined with apatinib and PD-1 inhibitors.

To our knowledge, this multicenter retrospective study included a larger sample size than did the aforementioned reports. This retrospective study showed that TACE-AP provides a more significant survival benefit than TACE-A in patients with advanced HCC. Zhu et al. (18) reported a median OS of TACE combined with lenvatinib and PD-1 inhibitors of 16.9 months (95% CI: 14.9–18.8), showing that this therapeutic regimen is more effective than TACE combined with lenvatinib, which is similar to our findings. Many studies have shown that TACE combined with targeted therapy and immunotherapy significantly improves the survival of patients with unresectable advanced HCC (19, 20). The most likely reason is that ischemic tumor necrosis due to TACE increases the release of tumor antigens, thereby increasing the expression of PD-1 and PD-L1, which improves tumor recognition (21, 22). The second possible reason is that the tumor microenvironment in advanced HCC is in an immunosuppressed state, leading to T cell dysfunction. Furthermore, the anti-VEGF effect of apatinib reduces immunosuppression in the tumor microenvironment, creating an inflammatory environment more suitable for T cell responses and an improved immunosuppressive state (23). In addition, the therapeutic drug for TACE in these studies was doxorubicin, which may induce and enhance immunogenic cell death and enhance immunity (24, 25). Therefore, the combination of TACE with apatinib and a PD-1 inhibitor may have a synergistic effect that achieves improved clinical efficacy in the treatment of patients with unresectable HCC.

Ju et al. (19) reported a median OS of patients treated with TACE combined with apatinib and camrelizumab of 24.8 months. A previous study by Cao et al. (20) on the treatment of unresectable advanced HCC showed a median OS and PFS of TACE combined with lenvatinib and sintilimab of 23.6 and 13.3 months, respectively. Liu et al. (26) reported a median OS and PFS of TACE combined with lenvatinib and camrelizumab in advanced HCC of 24 and 11.4 months, respectively. The results of TACE combined with targeted therapy were better than those of our study, which may be due to large differences in the baseline characteristics. Our study enrolled a greater number of patients with advanced-stage disease. The proportion of patients with BCLC stage C tumors enrolled in the above-mentioned studies was 76.8%, 75%, and 45%, respectively, but all patients enrolled in our study were BCLC stage C. Another possible reason is that the patients we enrolled had large tumor burdens with a maximum tumor diameter > 7 cm; therefore, it should be noted that the proportion of patients with PVTT invasion was as high as 50.5%, which may have greatly shortened the survival time of these patients. In addition, after the outbreak of COVID-19, due to the local epidemic prevention and control policies, some patients experienced delayed drug therapy, which may affect therapeutic efficacy. Nevertheless, compared with the above retrospective studies, the efficacy of TACE combined with apatinib and PD-1 inhibitors in advanced HCC patients reported in this study cannot be considered lower because of the relatively larger number of patients enrolled. Moreover, we used PSM to analyze the data, which reduced patient selection bias.

The incidence of TRAEs in this study was consistent with the published data (27–30). TACE-AP and TACE-A are safe, and the adverse reactions are tolerable. In this study, the adverse reactions related to embolism mainly included post-embolic syndrome (fever, nausea, vomiting, and pain) and apatinib-related adverse reactions, including hand-foot skin reactions, hypertension, proteinuria, fatigue, hoarseness, oral ulceration, and rash. The occurrences of hyperthyroidism, hypothyroidism, myocarditis, and pneumonia may be related to the use of PD-1 inhibitors. After symptomatic treatment or the temporary interruption of medication, these adverse event-related symptoms were relieved or eliminated. The incidence of TRAEs related to TACE and apatinib was not significantly different between the TACE-A and TACE-AP groups, suggesting that PD-1 inhibitors did not increase the risk of developing adverse effects in patients that underwent TACE. Therefore, our findings suggest that TACE combined with apatinib or TACE combined with apatinib and a PD-1 inhibitor to treat advanced HCC patients is controllable and safe.

This study was retrospective in nature. We achieved a balance in the baseline data of patients in the TACE-A and TACE-AP groups by using PSM analysis. There was a large reduction in sample size in the TACE-A group, which might have led to some key data not being included in the analysis, with analysis based on the differential baseline data lacking credibility for the control study. Therefore, to compensate for these shortcomings, we applied the stochastic survival forest model to further analyze the relevant factors affecting OS. Combined with traditional survival analysis, the final results showed that PVTT invasion, tumor number, AFP, TBIL, and treatment mode were continually the most significant prognostic factors affecting patient survival, consistent with previously reported research (8, 18).

This study has some limitations. First, this was a multicenter retrospective study, and the data were analyzed by PSM; however, potential patient selection bias could not be completely avoided. Second, multiple PD-1 inhibitors were used in this study, and the consistency of drug treatment could not be guaranteed. In addition, some patients did not receive apatinib or PD-1 inhibitors regularly after TACE due to economic or epidemic prevention and control measures. If patients had received combination therapy regularly, they may have obtained better clinical outcomes.

## Conclusion

In conclusion, our study shows that, compared with TACE combined with apatinib, TACE combined with apatinib and a PD-1 inhibitor significantly prolonged OS and improved the ORR in patients with advanced HCC. Furthermore, the observed treatment-related reactions were safe and controllable. Nonetheless, these findings should be confirmed by prospective, multicenter, randomized controlled trials.

## Data availability statement

The original contributions presented in the study are included in the article/ **Supplementary Material** . Further inquiries can be directed to the corresponding authors.

## Ethics statement

The studies involving human participants were reviewed and approved by The study was approved by the Ethics Committee of the Affiliated Cancer Hospital of Zhengzhou University and Henan Cancer Hospital review board (Approval number 2019198). Written informed consent for participation was not required for this study in accordance with the national legislation and the institutional requirements.

## Author contributions

Conception and design the study: H-LL, H-TH; Provision of study materials or patients: W-LX, G-SC, GW, W-JF, Q-JY. Collection and assembly of data: W-LX, Y-G, X-HZ, S-JX. Data analysis and interpretation: X-HZ, Y-G. Manuscript writing: W-LX. Manuscript reviewing: H-LL, H-TH. Final approval of manuscript: All authors.

## Funding

This work was supported by The National Natural Science Foundation (82002596), Henan Province Natural Science Foundation (212300410403), Medical Science and Technology Research Project of Henan Province (no. LHGJ20190633), Science and Technology Department of Henan Province (No. 212102310162), Beijing Health Alliance Charitable Foundation (HN-20201017-001), and the Technology Major Project of the Ministry of Science and Technology of China (2018ZX10303502).

## Acknowledgments

Thanks to all patients and medical staff who participated in the study.

## Conflict of interest

The authors declare that the research was conducted in the absence of any commercial or financial relationships that could be construed as a potential conflict of interest.

## Publisher’s note

All claims expressed in this article are solely those of the authors and do not necessarily represent those of their affiliated organizations, or those of the publisher, the editors and the reviewers. Any product that may be evaluated in this article, or claim that may be made by its manufacturer, is not guaranteed or endorsed by the publisher.
